# Diarrhœa

**Published:** 1890-09-06

**Authors:** 


### The Practitioner's Mirror and Retrospect.

*[The Editor will be glad to receive offers of co-operation and contributions from members of the profession. There is no doubt that much valuable material full of interest and instruction is at present lost to science. This is largely so in the case of (1) the younger and less-known members of the hospital staff (2) those connected with cottage hospitals, and (3) the great body of medical practitioners not attached to any institution. The co-operation of all is cordially invited to enable the Editor to make* THE HOSPITAL *of the utmost possible use and value to the profession. Specialists willing to review books on their special subjects are invited to communicate this fact at once. All letters should be addressed to* THE EDITOR, THE LODGE, PORCHESTER SQUARE, LONDON, W.]

### MEDICINE.

### DIARRHŒA.

The treatment of diarrhœa is certainly not one of those matters in which there is the concord which might be desired. This arises first, because diarrhœa is often merely a symptom of other maladies: and, secondly, because when there is no valid reason to regard it as a symptom of other maladies, different views are taken as to the exact causation. By being a symptom of other maladies we mean when diarrhœa occurs as a consequence of portal congestion, or of liver affection, or of dyspepsia, or in connexion with phthisis, or during enteric fever, &c. It is not, however, always easy to trace diarrhœa of this variety to its source, and not unfrequently the diarrhœa has been treated by astringents or otherwise when the cause of the flux should have been primarily attacked. By diarrhœa, without valid reason for regarding it as due to some other malady, we refer to that form of looseness which so often prevails in the autumnal season, both among children and adults, also to that variety which is so prevalent in hot climates, and from which so many who return from tropical climates suffer. Now one of the most common forms of diarrhœa in this country is simply due to portal congestion and its results, an overflow of acid bile into the intestines, and this might well be termed *diarrhœa biliosa.* It is marked by copious bilious stools passed with anal smarting and burning, and probably more or less griping abdominal pain. It may depend upon a course of too free living, or it may, and perhaps as often as not, does originate from cold or chill, which driving the blood from the surface into the interior tends to congest the liver. It is manifest that astringents are useless for such a diarrhœa, and that the proper course would be to prescribe some cholagogne medicine and so assist the liver in its attempts to relieve itself. Similarly when diarrhœa happens as a consequence of dyspepsia, and especially when the flux assumes a lienteric character, care in diet will probably be more efficacious than any kind of medicine. When, however, we have a diarrhœa to deal with for which no adequate cause can be assigned, treatment seems to depend very much on the peculiar views of the practitioner. It is only recently we were told by high authority that diarrhœa and especially summer diarrhœa is caused by a microbe, which having its habitat in the ground is conveyed into the intestines through the medium of water. And it was further advanced that this microbe is partial to sandy, porous soils, which, if correct, would change our views as to porous soils being more healthy than dense clay. But this microbe has not yet been isolated, and, therefore, we have not so much reason to believe in a microbe being the cause of diarrhœa, as we have that Koch's famous comma bacillus is the cause of that most severe form of flux, cholera. But it has also been advanced that the microbe is not really the cause of the diarrhœa, but that toxic excretions emitted by the microbes are the cause. The practitioner who believes in microbes causing diarrhœa would place faith in some germicide. The practitioner who believes in microbes emitting toxic excretions would look to the production of intestinal antisepsis. But there are others who believe in the excitation of diarrhœa by abnormal products of digestion giving rise to agencies toxic in their nature which irritate the intestines. During digestion active processes of fermentation undoubtedly take place by which toxic products are formed, but as a rule these are not present in sufficient quantity to irritate the bowels and are not absorbed into the blood. But it is advanced that without any of the ordinary symptoms of indigestion or dyspepsia this may take place. Recently, in the *Practitioner*, Dr. J. Michell Clarke, assistant physician to the Bristol Hospital, has published an article on the use of *hydronaphthol* in the treatment of diarrhœa. He says "in the treatment of intestinal catarrhs we should expect that the production of a state of antisepsis in the alimentary canal would cut short the disease." Hydronaphthol is generally prescribed in gelatine capsules, or simply suspended in milk. The dose is from two to three grains every two hours, for three to six doses, afterwards every three or four hours. For children under one year the dose is half a grain. Dr. Clarke recommends hydronaphthol in the diarrhœa of typhoid; in the diarrhœa of children with green coloured stools, and in dysenteric diarrhœa.

